# Blood does not buy goodwill: allowing culling increases poaching of a large carnivore

**DOI:** 10.1098/rspb.2015.2939

**Published:** 2016-05-11

**Authors:** Guillaume Chapron, Adrian Treves

**Affiliations:** 1 Grimsö Wildlife Research Station, Department of Ecology, Swedish University of Agricultural Sciences, Riddarhyttan 73091, Sweden; 2 Nelson Institute for Environmental Studies, University of Wisconsin, 30A Science Hall, 550 North Park Street, Madison, WI 53706, USA

**Keywords:** conservation, illegal hunting, policy signal, large carnivore, wolf

## Abstract

Quantifying environmental crime and the effectiveness of policy interventions is difficult because perpetrators typically conceal evidence. To prevent illegal uses of natural resources, such as poaching endangered species, governments have advocated granting policy flexibility to local authorities by liberalizing culling or hunting of large carnivores. We present the first quantitative evaluation of the hypothesis that liberalizing culling will reduce poaching and improve population status of an endangered carnivore. We show that allowing wolf (*Canis lupus*) culling was substantially more likely to increase poaching than reduce it. Replicated, quasi-experimental changes in wolf policies in Wisconsin and Michigan, USA, revealed that a repeated policy signal to allow state culling triggered repeated slowdowns in wolf population growth, irrespective of the policy implementation measured as the number of wolves killed. The most likely explanation for these slowdowns was poaching and alternative explanations found no support. When the government kills a protected species, the perceived value of each individual of that species may decline; so liberalizing wolf culling may have sent a negative message about the value of wolves or acceptability of poaching. Our results suggest that granting management flexibility for endangered species to address illegal behaviour may instead promote such behaviour.

## 1. Introduction

Most governments have a legal duty to conserve and restore endangered wild fauna and flora species [1] for the benefit of current and future generations [2]. The conservation of biodiversity can be controversial as it often imposes limits to human activities [3] and negative actions—such as environmental crimes—need to be contained at levels that do not preclude conservation successes. Evaluating the effectiveness of interventions aimed at abating environmental crimes has become fundamental to conservation policy making. For wildlife populations, environmental crimes include illegal hunting or poaching. However, while identifying the causes and extent of mortality is a central line of inquiry in biology and ecology, it remains notoriously difficult for poaching because evidence is typically concealed from enforcement agencies and scientists alike. As a consequence, illegal hunting or poaching has become a major cause of concern for the conservation of endangered species, particularly for controversial species such as large carnivores [4]. The few available quantitative studies have revealed strong effects of poaching on carnivore demography [5–7]. Poaching accounted for more mortality events than any other cause in the reintroduced populations of the red wolf *Canis rufus* [8] and more than half of the total mortality of Mexican grey wolves *C. lupus baileyi* [9]. In a unique, large but closed population, poaching accounted for half of the mortality of grey wolves in Scandinavia and two-thirds of poaching remained undetected using direct methods of observation [10]. Poaching has also significantly contributed to the extinction of a reintroduced brown bear (*Ursus arctos*) population in Austria—the first modern time extinction of a large carnivore population in the European Union [11]—and the quasi-extinction of wolves in Southern Spain [12].

**Figure 1.**
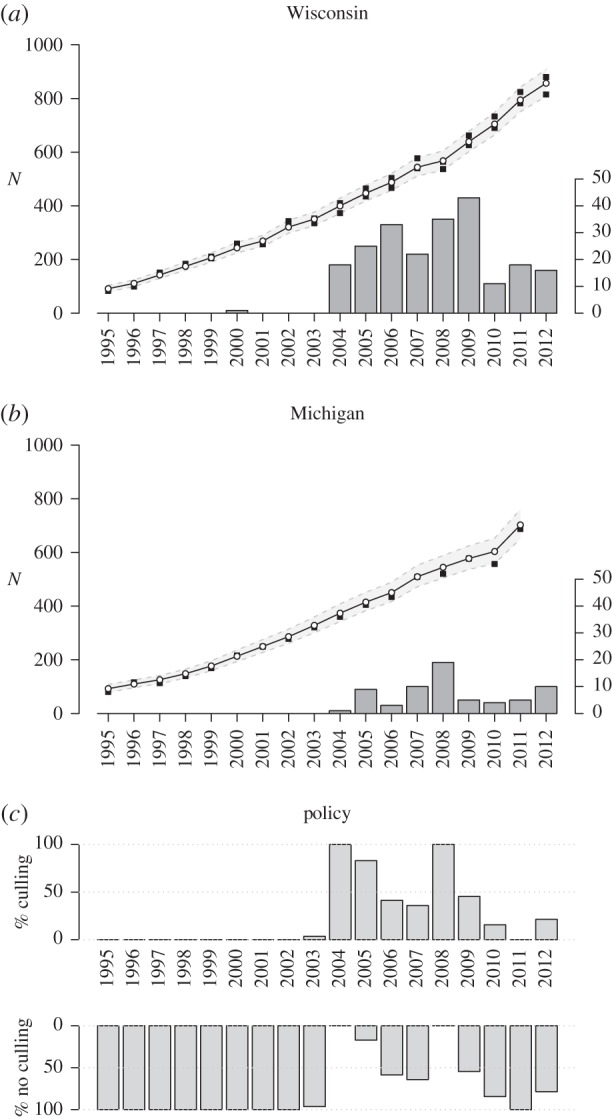
Wolf population history in Wisconsin (top) and Michigan (middle) and policies (bottom). The black squares are FWS population counts (scale on left axis, minimum and maximum for Wisconsin, minimum for Michigan), the grey area is the 95% credible interval of the fitted population model, the histogram shows the number of wolves culled (scale on right axis). The bottom panel shows the proportion of each year in which culling was allowed (or not). Some wolves were killed legally when culling was not allowed (e.g. year 2011) because the FWS allows killing individuals of an endangered species ‘to protect himself or herself, a member of his or her family, or any other individual from bodily harm’ (ESA §11(a)(3)).

Quantifying the variation in poaching and especially how it responds to policy changes has become one of the most crucial questions for the conservation of large carnivores [6,13,14]. One proposal to address poaching of carnivores has been to legalize or liberalize killing. The difficulty of obtaining evidence about poaching has provided fertile ground for the notion that poachers will refrain if legal recourse exists, such as government-sponsored culling or regulated, public hunting [7,15,16]. In a review of conservation conflicts, Redpath *et al*. [17] argued that strict protections would need to be made more flexible to achieve more durable conservation outcomes. Woodroffe & Redpath [18] further insisted that ‘Pragmatic conservationists have long recognized that allowing some predator control—whether or not it achieves its stated aims—can help to build tolerance among land managers who might otherwise block conservation efforts’ albeit without providing any quantitative evidence. Despite this assumption remaining largely untested, or contrary predictions that legalizing killing stimulates intentions to poach [14], the notion has been promoted by several governments and management authorities, see e.g. Swedish Government Official Reports [19]. In 2007, the US Fish and Wildlife Service (FWS) proposed removing federal protections (delisting) for Yellowstone grizzly bears by claiming that ‘a future hunting season also may increase tolerance and local acceptance of grizzly bears and reduce poaching in the GYA’ [20]. It, however, later acknowledged in the final rule that ‘there is no scientific literature documenting that delisting would or could build…tolerance for grizzly bears' but nevertheless maintained that ‘effective nuisance bear management benefits the conservation of the Yellowstone grizzly bear population by … minimizing illegal killing of bears' [21]. Despite no additional scientific evidence being produced, the USFWS makes the same claims in 2016 in its new proposed rule to delist the Yellowstone grizzly bear [22] and argues that ‘while lethal to the individual grizzly bears involved, these removals promote conservation of the GYE grizzly bear population by minimizing illegal killing of bears and promoting tolerance of grizzly bears'. Similar claims have been made in court for legalizing wolf culling. For example, the FWS asserted in a federal court that ‘in the absence of adequate measures to control known depredating wolves, … individuals will resort to illegal killing’ and the Swedish Ministry of the Environment replied to an infringement procedure by the European Commission that ‘The Swedish Government and the Swedish Parliament considers that a [sic] limited and strictly controlled license hunting is needed to obtain local acceptance’ of wolf conservation [23]*.* In winter 2015–2016, a wolf hunt in Finland (with a quota of 46 out of 250 wolves) was justified by Finnish authorities by the claim that ‘The purpose of derogations granted to manage the population is to respond to the views put forward in the wolf territories and to develop a legal operating model of population management for dealing with disruptive individuals, and thus preventing illegal killing of wolves’ [24]. The claim that legalizing culling or hunting will reduce poaching has become a fundamental issue for the conservation and management of large carnivores in human-dominated landscapes. It has often been discussed but never properly evaluated and is still made by authorities to justify substantial culling of recovering and still fragile populations. In this paper, we took advantage of replicated quasi-experimental changes in wolf policies in two US states (Wisconsin and Michigan, figure 1) to assess whether liberalizing culling of wolves changed wolf population dynamics from 1995 to 2012.

## 2. Material and methods

The policy changes were replicated over six treatments in each state (when states had legal authority to cull) interspersed with six control periods (states did not have that authority following federal court decisions). Decisions to kill wolves by the two states were independent. We used a Bayesian hierarchical model to estimate variations in wolf population growth rates as a function of policy changes.

### (a) Annual wolf population counts

Wolves were extirpated from both states in the 1950s, then recolonized Wisconsin by 1978 without direct human intervention, probably from Minnesota [25]. Wolves recolonized Michigan by 1989, probably from Wisconsin [26]. From 1979 to April 2003, Wisconsin wolves were classified as federally endangered (listed) but the classification changed in the following years as we explain further below [15]. The Wisconsin population grew from 0 to a minimum of 815 by April 2012 and the Michigan population grew from 0 to 587 by April 2011 (no census was conducted in 2012). We defined a wolf-year *t* as starting 15 April of year *t* − 1 and ending 14 April of year *t*. We obtained state wolf population estimates for each wolf-year 1995–2012 (minimum counts for Wisconsin and Michigan, maximum counts for Wisconsin) [27–29]. We obtained culling data and the variables used in density dependence from annual reports issued by Wisconsin (www.dnr.wi.gov (accessed September 2012) and through a memorandum of understanding with A.T., MoU MSN146937). We obtained data for Michigan from a state carcass tracking database accessed by the Little River Band of Ottawa Indians, which provided it to A.T., through a federal Consent Decree. The methods used for data collection were described previously [26,30].

### (b) Wolves killed

To avoid bias resulting from censored or missing data, only the completely reported wolf mortality was treated as culling in our population model. Two circumstances increased our confidence in complete reporting of culling data. First, all culling was ordered or permitted to agents by the states of Wisconsin and Michigan. Second, by law, all wolf killing had to be reported by the government agencies during the study period [31]. We counted only those wolves killed by government-approved permits following verified or perceived threats to livestock, pets or human safety (the latter could occur during periods in which other culling was not allowed, electronic supplementary material, table S1). The signal associated with the onset of culling was far less ambiguous than that associated with delisting due to agency announcements and rule publications preceding the official date of delisting [15].

### (c) Reproduction of packs

We estimated reproductive performance of packs using a binary variable (0 for no reproduction or 1 for reproduction). Estimates of reproductive performance of wolf packs were sometimes made *post hoc* after field observation of changes in pack size, sometimes from summer howling surveys that elicited pup responses, and sometimes from aerial radio-telemetry flights that included visual detection of young individuals in packs.

### (d) Area occupied by wolf packs

For the wolf-years 2000–2011, we estimated total area occupied by wolf packs each year using the ArcGIS® spatial geometry calculator and polygon shapefiles from data provided by the WI DNR to A.T. under MoU MSN146937, which stipulated confidentiality of geographical locations of wolf packs.

### (e) Management authority

On 1 April 2003, the FWS temporarily reclassified wolves as threatened (a lower level of protection under federal authority), which gave the states the authority under the Endangered Species Act (ESA) rule 4(d) to kill wolves implicated in verified damage to property (culling) [15]. In the ensuing years, federal courts and the FWS changed wolf classification to endangered (listed), removed federal protection (delisted), or separately gave the states sub-permits to cull wolves despite being listed as endangered (i.e. ESA 10(a)(1)(A) permits). Courts rescinded those sub-permits in both cases after variable intervals during which wolves were culled. As a result, the proportion of days in which culling was legally permitted under state authority was not equal to the proportion of days in which wolves were delisted. There were 12 treatment periods as a result of the policy changes (electronic supplementary material, table S1 and figure 1, bottom panel). Although the two states underwent nearly identical calendars of authority (electronic supplementary material, table S1), they managed wolves independently including independent contracts with federal Department of Agriculture (USDA) regional offices to cull wolves. There was no coordination of implementation between the two states [31], other than communicating the removal of radio-collared wolves that originated in the other state.

### (f) Population-policy model

For each state *S* (Wisconsin or Michigan) at time *t*, the true population size 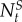 followed a lognormal distribution of the deterministic population size 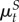 with a stochastic process error *σ*_proc_ on the log scale having a weakly informative prior (electronic supplementary material, table S2):
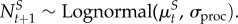


The deterministic model was exponential and included the number of wolves culled 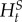 with a parameter *γ* allowing for compensation or depensation (super additivity) given an informative prior from North American data [32]
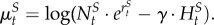


Growth rate 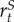 was modelled as a linear function of the number of days that culling was allowed 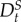 in state *S* during year *t* (the policy signal):
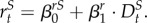


Coefficients of the linear function were parametrized with non-informative priors (electronic supplementary material, table S2).

We modelled the observed minimum counts of annual wolf population size 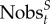 by a Poisson distribution with a mean 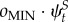, with 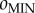 having a non-informative prior on [0,1] to consider that a minimum count underestimates population size and 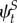 itself drawn from a Gamma distribution with mean equal to the prediction of the process model and a standard deviation 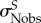 for observation error having a weakly informative prior:
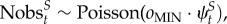

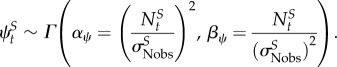


We followed the same approach for the observed maximum counts in Wisconsin modelled by a Poisson distribution with a mean 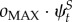, with 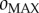 having a non-informative prior on [1,10] to consider that a maximum count likely overestimates population size.

### (g) Density dependence on pack size

Using data from all packs monitored in Wisconsin from 1995 to 2011 (data from 2012 were not available), we modelled the true size *P_i_* of each pack *i* at time *t* as being Poisson distributed with mean 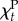 being a linear function of population size 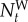 (W for Wisconsin) during year *t*:
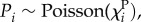

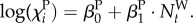
Coefficients of the linear function were parametrized with non-informative priors (electronic supplementary material, table S2).

Observed size Pobs*_i_* of each pack followed a gamma distribution with mean equal to the prediction of the process model of pack size and a standard deviation for observation error 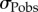 having an informative prior assuming an error of ±1 wolf when monitoring pack size (electronic supplementary material, table S2):
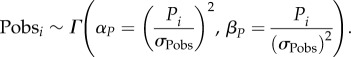


### (h) Density dependence on probability a pack reproduces

Using data from all packs monitored in Wisconsin from 1995 to 2011, we modelled the event of a pack reproducing as following a Bernoulli distribution with probability described by a logistic function of population size 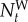 (W for Wisconsin) during year *t*:
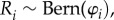

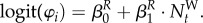


Annual coefficients of the linear function were parametrized with non-informative priors (electronic supplementary material, table S2).

### (i) Density dependence on area occupied by packs

Using wolf pack territory sizes [30] from 2000 to 2011 (mapping prior to 2000 was not based on GPS locations), we calculated the total area (in square kilometres) occupied by wolf packs in year *t*. In the hierarchical model, we then assumed this area was a linear function of population size 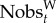 (W for Wisconsin) at year *t* with a stochastic error 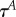:
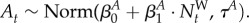
Coefficients of the linear function were parametrized with non-informative priors (electronic supplementary material, table S2).

### (j) Monte Carlo Markov chain inference

We ran eight Monte Carlo Markov chains (100 000 iterations thinning by 10 after adapting and updating for 50 000 iterations) in R [33] with JAGS [34]. We checked convergence with the Gelman & Rubin [35] and Heidelberger & Welch [36] diagnostic tests. Posterior parameter estimates revealed a lack of density dependence (electronic supplementary material, table S2), and if any density dependence had occurred, it was much more likely to be positive (electronic supplementary material, figure S1–S4).

## 3. Results

**Figure 2.**
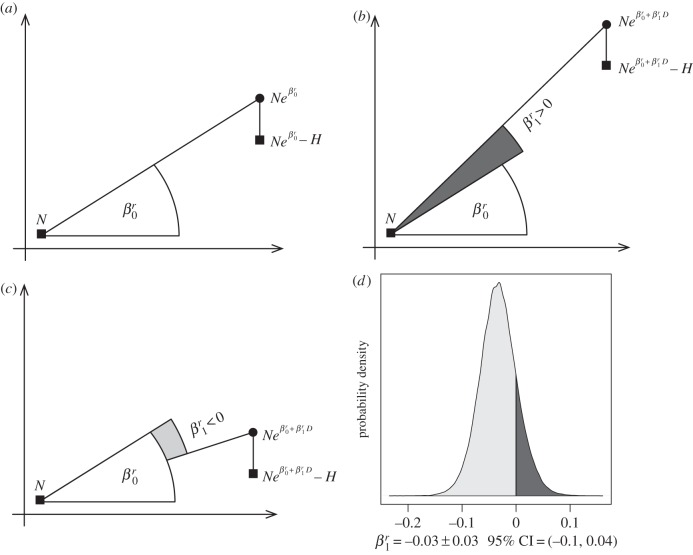
Conceptual model of how culling policy signal influences growth rate. From one time step to the next (horizontal axis), a population has a potential growth rate 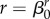 which does not account for the animals culled *H* (panel *a*). With a culling policy signal lasting duration *D* (proportion of a year), the potential growth rate becomes 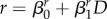, and increases when 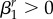 (through a decrease of poaching, panel *b*), or alternately decreases when 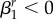 (through an increase of poaching, panel *c*) as we found here. The effect 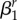 of the culling policy signal on population growth rate *r* is independent of the number of wolves culled *H* during implementation. The posterior density distribution 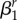 (panel *d*) shows a decline of growth rate is five times more likely 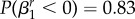 (light grey area) than an increase 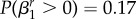.

We found that the policy signal generated by liberalizing wolf culling was associated with an average decrease in wolf potential population growth rates. With no culling policy signal, the annual potential growth rate (excluding the culled wolves, figure 2) was *r* = 0.16 ± 0.02, 95% CI = 0.12–0.2 in Wisconsin (*r* = 0.14 ± 0.02, 95% CI = 0.1–0.18 in Michigan). However, with a year-long culling policy signal, we found annual growth rate had a 83% probability to be lower (figure 2*d*) with *r* = 0.12 ± 0.03, 95% CI = 0.07–0.19 in Wisconsin (*r* = 0.10 ± 0.03, 95% CI = 0.05–0.17 in Michigan). Crucially, this decrease in population growth was independent of the number of wolves culled, as our population model made the explicit distinction between a policy (number of days when culling was allowed) and its implementation (number of wolves culled, figure 2). The model could therefore detect an effect of allowing culling even if no wolves were killed.

Similar results emerged when we replaced the culling policy signal with the announcement of federal delisting as the policy signal because culling happened primarily when wolves in the two states were federally delisted (figure 1). Even though we cannot disentangle the two causal mechanisms (allowing culling or delisting), our analyses suggest a policy signal to relax protections for wolves affected subsequent wolf population growth.

## 4. Discussion

We infer that variations in wolf population growth rates we detected were variations in poaching resulting from policy changes. Although our model does not include poaching as an explicit parameter, poaching was the most parsimonious explanation for observed decrease in wolf population growth rates, because we could rule out alternative plausible biological explanations. The most intuitive explanation of slowing growth with a growing population would be negative density dependence. We could not directly include density dependence in our population prediction model as it would be a weakly identifiable parameter with poaching. Instead, we used additional data in a Bayesian model that were biologically meaningful to detect density dependence (average pack size, probability a pack reproduces, and area occupied by wolf packs, see electronic supplementary material, table S2 and figures S1–S4). As with prior studies on Wisconsin's wolf population [28], we did not detect any negative density dependence. A second plausible alternative explanation for the observed decrease in population growth rates would be super-additive mortality, i.e. the decline in growth rates we detect might reflect other wolves dying because of the loss of wolves killed during culling periods. The debate whether human-induced mortality in wolves is compensatory, additive or super-additive is not settled yet [32,37,38]. We therefore used an informative prior by assuming the same additivity as found for wolf populations across North America [32] (see electronic supplementary material, table S2). If the decline in growth rates we detected had been caused by super-additive mortality, that mortality would need to be stronger than any reported before. We consider such strong super-additive mortality unlikely because culling was implemented by springtime and summertime live-trapping principally [31] and not by hunting chase or other methods that might disturb entire packs during sensitive reproductive periods. Third, wolf emigration to neighbouring states was unlikely to increase only by a policy announcement. Fourth, natural fluctuations in wolf population size and monitoring quality were accounted for by our process and observation errors [39]. The increase of poaching we infer is therefore unlikely to be a consequence of a failure to account for natural fluctuations, which would in addition likely be less important than seen in smaller populations [39]. Finally, because periods without culling were directly inversely correlated with periods with culling [40], one could argue instead that the court-ordered termination of culling permits had triggered ‘frustration poaching’. However, such frustration measured as negative attitudes to wolves was present well before the first culling was permitted [41], as was the poaching that might be caused by frustration and penalties for wolf poaching did not change [15]. In addition, a quasi-experimental longitudinal study of attitudes to wolves before and after Wisconsin's October 2012 regulated, public hunt of wolves revealed a decline in tolerance among men with familiarity with hunting who lived in Wisconsin's wolf range, exactly opposite to the predicted decrease in ‘frustration’ with more liberalized wolf killing [42]. Studies of attitude change since 2001 have repeatedly shown that liberalizing wolf killing did not reduce inclination to poach among residents of Wisconsin's wolf range [43,44]. As none of the alternative explanations had statistical or biological support, we could infer that variations in growth rates we detected were variations in poaching resulting from policy changes.

Our approach is different from previous studies [10,28,40] because we do not aim to quantify total poaching rate and its variations. Because the two states' wolf populations were not closed, migration rates were unknown and the cryptic nature of poaching events for radio-marked animals precluded obtaining informative parameter estimates. Our model instead estimated how poaching responded to an annual policy signal, without estimating total poaching, and it treated separately the policy signal from its implementation, which were only weakly correlated. Our results are also consistent with empirical studies that link intentions to poach with culling policy. For example, studies in Wisconsin that measured intention to poach wolves found those intentions rose in parallel with liberalized culling [44] and those intentions did not decline after a period with liberalized culling [43]. Moreover, legalizing wolf-hunting led to a continued decline in tolerance for wolves by summer 2013 [42]. We hypothesize the legal opening of an additional source of mortality sent a signal that the net benefits of wolves had declined, consistent with psychological theory of hazard assessment. For example, a recent experimental study of messaging found that public acceptance of American black bears *Ursus americanus*, diminished when informational messages did not include benefits of bears [45]. When the government kills a protected species, the perceived value of each individual of that species may decline. Liberalizing wolf culling may have sent a negative message about the value of wolves or that poaching prohibitions would not be enforced.

The assumption that legal killing would decrease illegal killing has often been portrayed as an effective way to manage recovering large carnivore populations and, despite no prior scientific evaluation, has been promoted by some conservation authorities [46]. For example, the World Conservation Union—IUCN claims through its manifesto for large carnivore conservation in Europe that ‘legalised hunting of large carnivores can be a useful tool in decreasing illegal killing’ [47]. In light of our results, we find this recommendation has no support. Indeed, liberalizing killing appears to be a conservation strategy that may achieve the opposite outcome than that intended.

Because the wolf habitat in the two US states in our study does not include wilderness and consists mostly of a human-dominated matrix, our results are particularly meaningful to understand the mechanisms of coexistence between large carnivores and people worldwide [48,49]. We recommend that efforts at leniency in environmental protections are not justified as a way to prevent illegal activities unless solid rigorous evidence is provided. We conclude by stressing that many environmental policies produce both signals and implementations, which can be treated as experimental interventions with separate and possibly contradictory effects. Whether anti-pollution or anti-poaching policies are being crafted, the perception of that policy may be as important to understand carefully, as are the enforcement and compliance checks that represent implementation.

## Supplementary Material

Supplementary Material

## Data accessibility

The datasets supporting this article are available at http://dx.doi.org/10.5061/dryad.b7d7v.
