# Correction to ‘Blood does not buy goodwill: allowing culling increases poaching of a large carnivore’

**DOI:** 10.1098/rspb.2016.2577

**Published:** 2016-12-28

**Authors:** Guillaume Chapron, Adrian Treves

*Proc. R. Soc. B* **283**, 20152939. (2016; Published online 11 May 2016) (doi:10.1098/rspb.2015.2939)

We recently discovered an error in [1] due to a misalignment of rows between columns in the dataset. Specifically, we misaligned by 1 year the population size with the number of wolves culled and the policy signal. The correct results are slightly different than the ones we presented: the effect we report becomes slightly stronger and some parameters see minor adjustments of their posterior values. The conclusion of our paper is still supported by the correct results.

The correct results indicate that with no culling policy signal, the annual potential growth rate was *r* = 0.17 ± 0.02 95% credible interval (CI) = 0.13–0.21 in Wisconsin (*r* = 0.15 ± 0.02 95% CI = 0.11–0.19 in Michigan). However, with a year-long culling policy signal, we found annual growth rate had a 92% probability to be lower (figure 1 in this article) with *r* = 0.12 ± 0.03 95% CI = 0.06–0.18 in Wisconsin (*r* = 0.10 ± 0.03 95% CI = 0.04–0.16 in Michigan). Corrected prior and posterior values for all model parameters are given in table 1. In the electronic supplementary material, we provide a commented R code with both the mis-aligned and the properly aligned datasets so that the reader can replicate both the original results and the corrected ones. Running this code requires the software JAGS [2] with the package R2jags [3].

**Figure 1.**
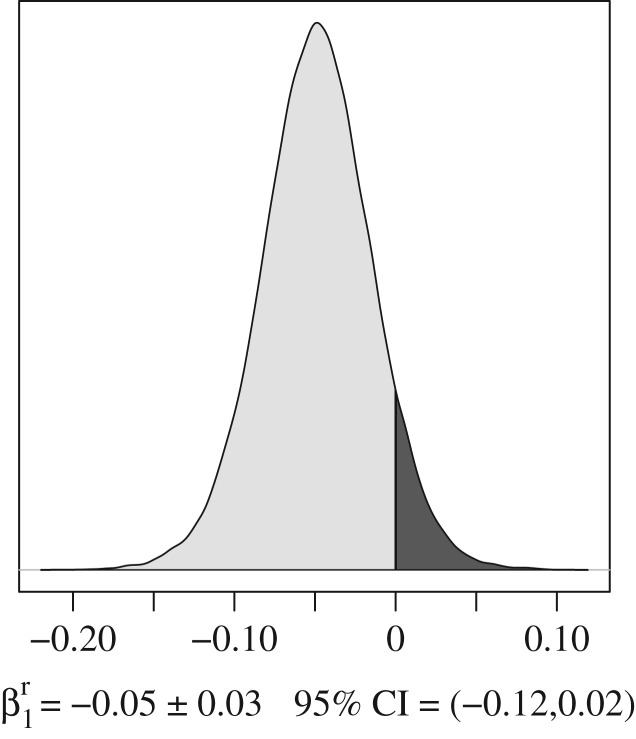
The posterior density distribution 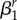 shows a decline of growth rate is 12 times more likely 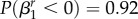 (light grey area) than an increase 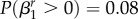 (dark grey area).

**Table 1.** Prior and posterior values for the dynamic model parameters.

prior choice	posterior distribution	
	median ± s.d.	95% credible interval
*population dynamic*		
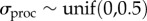	0.06 ± 0.02	0.03–0.09
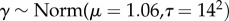	1.06 ± 0.07	0.92–1.2
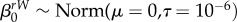	0.17 ± 0.02	0.13–0.21
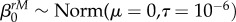	0.15 ± 0.02	0.11–0.19
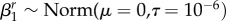	−0.05 ± 0.03	−0.12–0.02
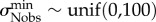	4.38 ± 3.3	0.17–12.29
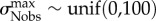	5.53 ± 4.4	0.23–16.42
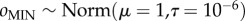 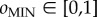	0.97 ± 0.02	0.93–1
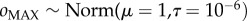 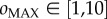	1.03 ± 0.02	1–1.08
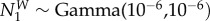	91.10 ± 6.15	79.43–103.57
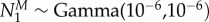	92.06 ± 7.4	78.15–107.39

Two other typographical errors were not detected during the proof process. 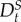 was the proportion (and not the number) of days that culling was allowed in state *S* during year *t*. The equation describing area as a linear function of population size should indicate we took the logarithm of area: 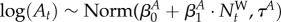 which explains the very small (but positive) values for 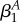.

## Supplementary Material

R code of the dynamic model
