# Supplementary material for: Blood does not buy goodwill: allowing culling increases poaching of a large carnivore
Source: Proc Biol Sci. 2016 May 11;283(1830):20152939. doi: 10.1098/rspb.2015.2939 (PMC4874699; doi:10.1098/rspb.2015.2939)
Supplement: Supplementary Material [file rspb20152939supp1.pdf]

Supplementary Information

Table S1. Periods for culling policy signals in both states, derived from *(12, 39)*, ESA sec. 10(a)(1)(A) and Humane Society of the U.S. et al. v. Jewell (U.S. District Court, D.C., 1:13-cv-00186-BAH Document 52, 2014.

Period start (dd/mm/yyyy)	Period end (dd/mm/yyyy)	Federal status	Culling**
15/04/1994	31/03/2003	Listed as endangered	not allowed
01/04/2003	30/01/2005	Down-listed to threatened	allowed
31/01/2005	31/03/2005	Relisted	not allowed
01/04/2005	13/09/2005	Sub-permit for culling issued	allowed
14/09/2005	23/04/2006	Sub-permit rescinded	not allowed
24/04/2006*	31/07/2006	Sub-permit for culling issued	allowed
01/08/2006	11/03/2007	Sub-permit rescinded	not allowed
12/03/2007	28/09/2008	Delisted	allowed
29/09/2008	03/05/2009	Relisted	not allowed
04/05/2009	30/06/2009	Delisted	allowed
01/07/2009	26/01/2012	Relisted	not allowed
27/01/2012	14/04/2012	Delisted	allowed

*States identical except sub-permit issuance on 6 May 2006 to Michigan instead of issuance on 24 April 2006 to Wisconsin *(12)*.

** Killing a wolf that posed a threat to human safety was always allowed under ESA sec.11(a)(3)

Table S2. Prior and posterior values for all model parameters.

**Prior choice**	**Posterior distribution**	
	Median ± SD	95% credible interval
Population dynamic		
$\sigma_{proc} \sim \text{unif}(0,0.5)$	0.06 ± 0.02	0.03 – 0.1
$\gamma \sim \text{Norm}(\mu = 1.06, \tau = 14)$	1.06 ± 0.07	0.92 – 1.19
$\beta_0^{rW} \sim \text{Norm}(\mu = 0, \tau = 10^{-6})$	0.16 ± 0.02	0.12 – 0.2
$\beta_0^{rM} \sim \text{Norm}(\mu = 0, \tau = 10^{-6})$	0.14 ± 0.02	0.1 – 0.18
$\beta_1^{r} \sim \text{Norm}(\mu = 0, \tau = 10^{-6})$	-0.03 ± 0.03	-0.1 – 0.04
$\sigma_{Nobs}^{\min} \sim \text{unif}(0,100)$	3.82 ± 3.39	0.19 – 12.63
$\sigma_{Nobs}^{\max} \sim \text{unif}(0,100)$	4.72 ± 4.5	0.23 – 16.78
$o_{MIN} \sim \text{Norm}(\mu = 1, \tau = 10^{-6})$ $o_{MIN} \in [0,1]$	0.97 ± 0.02	0.93 – 1
$o_{MAX} \sim \text{Norm}(\mu = 1, \tau = 10^{-6})$ $o_{MAX} \in [1,10]$	1.03 ± 0.02	1 – 1.08
Density dependence on pack size		
$\beta_0^{P} \sim \text{Norm}(\mu = 0, \tau = 10^{-6})$	1.17 ± 0.03 *	1.1 – 1.23
$\beta_1^{P} \sim \text{Norm}(\mu = 0, \tau = 10^{-6})$	0 ± 0	0 – 0
$\sigma_{Pobs} \sim \text{unif}(0,1)$	0.17 ± 0	0.16 – 0.17

Density dependence on probability a pack reproduces		
$\beta_0^R \sim \text{Norm}\left(\mu = 0, \tau = 10^{-6}\right)$	0.39 ± 0.13	0.14 – 0.64
$\beta_1^R \sim \text{Norm}\left(\mu = 0, \tau = 10^{-6}\right)$	0 ± 0	0 – 0
Density dependence on area occupied by packs		
$\tau^A \sim \text{T}\left(\alpha = 10^{-6}, \beta = 10^{-6}\right)$	97 ± 46	33 – 212
$\beta_0^A \sim \text{Norm}\left(\mu = 0, \tau = 10^{-6}\right)$	9.61 ± 0.1	9.42 – 9.81
$\beta_1^A \sim \text{Norm}\left(\mu = 0, \tau = 10^{-6}\right)$	0 ± 0	0 – 0

* on the log scale

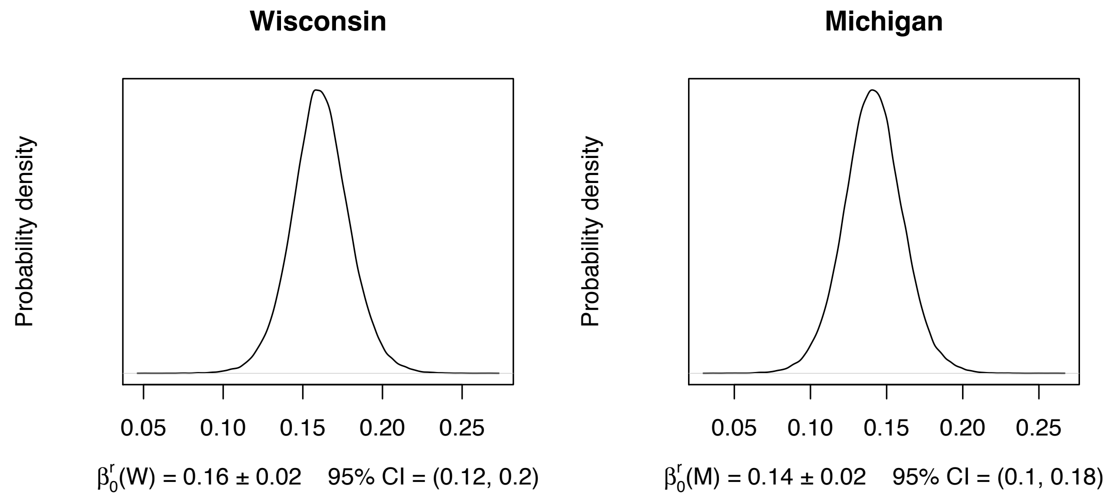

Figure S1. Posterior density distributions of intercepts β_0^{rW} (Wisconsin, left) and β_0^{rM} (Michigan, right) of population growth rates (without policy signal).

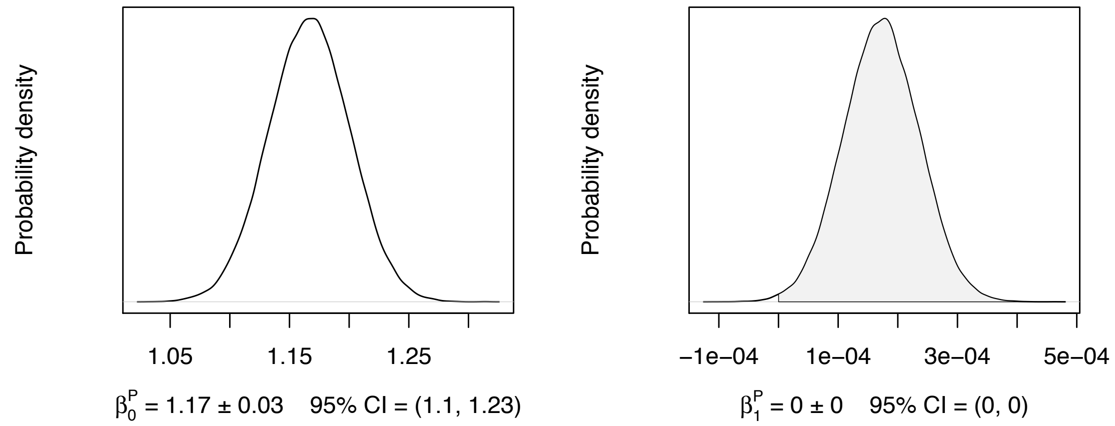

Figure S2. Posterior density distributions of linear model coefficients (intercept β_0^P) and (slope β_1^P) for pack size. The grey area under the curve at right indicates the probability there is no negative density dependence.

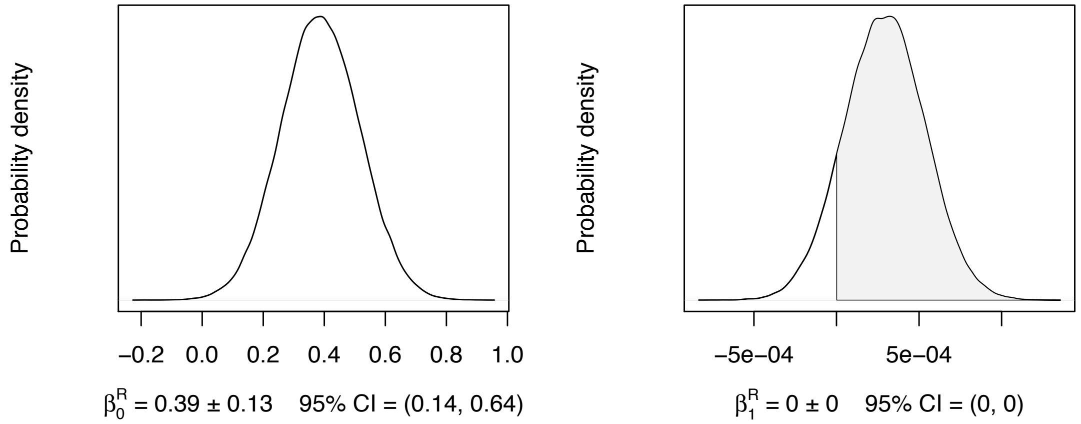

Figure S3. Posterior density distributions of linear model coefficients (intercept β_0^R) and (slope β_1^R) for the probability a pack reproduces. The grey area under the curve at right indicates the probability there is no negative density dependence.

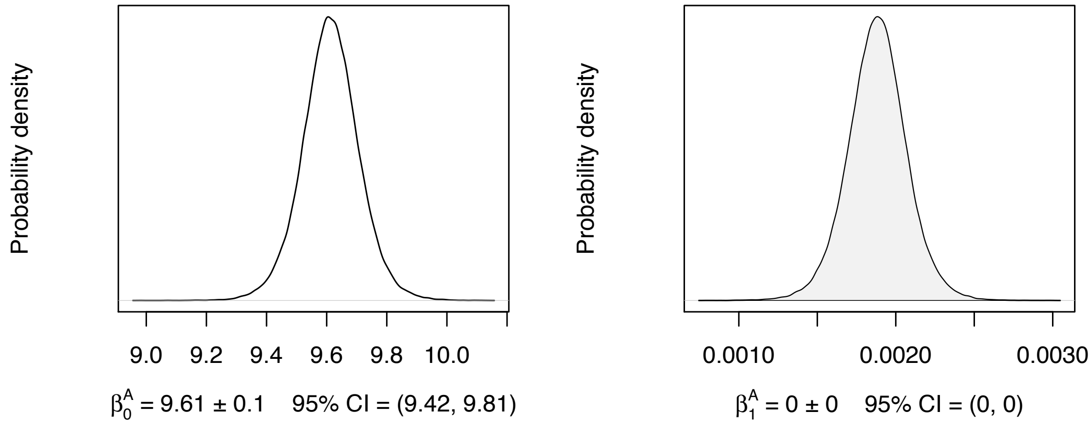

Figure S4. Posterior density distributions of linear model coefficients (intercept β_0^A) and (slope β_1^A) for area occupied by wolf packs. The grey area under the curve at right indicates the probability there is no negative density dependence.
